# Detection and management of postoperative atrial fibrillation after coronary artery bypass grafting or non-cardiac surgery: a survey by the AF-SCREEN International Collaboration

**DOI:** 10.1007/s11739-025-03861-2

**Published:** 2025-02-08

**Authors:** Giuseppe Boriani, Jacopo F. Imberti, William F. McIntyre, Davide A. Mei, Jeff S. Healey, Renate B. Schnabel, Emma Svennberg, A. John Camm, Ben Freedman

**Affiliations:** 1 Cardiology Division, Department of Biomedical, Metabolic and Neural Sciences, University of Modena and Reggio Emilia, Policlinico Di Modena, Via del Pozzo, 71, 41124 Modena, Italy; 2 Clinical and Experimental Medicine PhD Program, University of Modena and Reggio Emilia, Modena, Italy; 3 Population Health Research Institute, McMaster University, Hamilton, ON Canada; 4 Department of Cardiology, University Heart and Vascular Center Hamburg-Eppendorf, Hamburg, Germany; 5 German Center for Cardiovascular Research (DZHK), Partner Site Hamburg/Kiel/Lübeck, Hamburg, Germany; 6 Department of Medicine, Karolinska Institutet, Karolinska University Hospital, Huddinge, Stockholm, Sweden; 7 Cardiology Clinical Academic Group, City St George’s University of London, London, UK; 8 Heart Research Institute, Charles Perkins Centre, The University of Sydney, Sydney, Australia; 9 Department Cardiology, Concord Repatriation General Hospital, Hospital Road, Concord, Sydney, NSW 2139 Australia

**Keywords:** Acute care, Atrial fibrillation, Cardiac surgery, Postoperative atrial fibrillation, Stroke, Wearable devices

## Abstract

We developed a survey to describe current practice on the detection and management of new-onset postoperative atrial fibrillation (POAF) occurring after coronary artery bypass grafting (CABG) or non-cardiac surgery. We e-mailed an online anonymous questionnaire of 17 multiple choice or rank questions to an international network of healthcare professionals. Between June 2023 and June 2024, 158 participants from 25 countries completed the survey. For CABG patients, 62.7% of respondents reported use of telemetry to detect POAF on the ward until discharge, and 40% reported no dedicated methods for monitoring AF recurrences during follow-up. The largest number (46%) reported prescribing oral anticoagulants (OACs) at discharge if patients were at risk according to CHA_2_DS_2_-VASc/CHA_2_DS_2_-VA scores, and the most common duration of OAC therapy was 3 months to 1 year (43%). For non-cardiac surgery patients, POAF detection methods varied, with 29% using periodic 12-lead ECG and 27% using telemetry followed by periodic ECGs. For monitoring AF recurrence, 33% reported planned cardiology visits with ECG. Regarding OAC prescription during follow-up, 51% reported they prescribe OACs only for patients who are at risk of stroke, and 42% prescribe OACs for an interval of 3 months to 1 year. The most commonly reported barrier to OAC prescription was the lack of randomized controlled trial data. For both CABG and non-cardiac surgery, the reported methods for POAF detection and recurrences monitoring were heterogeneous and prescription patterns for OACs varied greatly. The most frequently reported concern about long-term anticoagulation was lack of randomized data, indicating the urgent need for sound studies that inform daily clinical practice.

**Supplementary Information:**

The online version contains supplementary material available at 10.1007/s11739-025-03861-2.

## Introduction

New-onset postoperative atrial fibrillation (POAF) is the most common complication following cardiac surgery and is also a frequent complication after non-cardiac surgery [1, 2]. Its incidence varies depending on the type of surgery, patient risk profile, and method of atrial fibrillation (AF) detection (e.g., continuous vs opportunistic ECG monitoring) [3–6]. POAF can be detected in approximately 30% of patients undergoing cardiac surgery, with the highest incidence observed in valve replacements (up to 50%), followed by aortic surgery (30%), and coronary artery bypass grafting (CABG) (20%) [3]. The pathogenesis is multifactorial, but direct cardiac tissue manipulation during surgery likely plays a key role. In contrast, POAF is detected in 0.4%–15% of patients undergoing non-cardiac surgery [7], potentially due to stressors acting on a predisposed substrate [3]. Rates are highest in patients undergoing non-cardiac thoracic surgery (7.5%) [8]. In both contexts, POAF is associated with worse outcomes, including prolonged hospital stay, stroke, myocardial infarction, heart failure, and mortality [3, 9–13]. A meta-analysis of 35 studies involving 2,458,010 patients found that POAF was associated with a higher risk of early stroke (odds ratio [OR]: 1.62; 95% confidence interval [CI] 1.47–1.80), early mortality (OR: 1.44; 95% CI 1.11–1.88), long-term stroke (hazard ratio [HR]: 1.37; 95% CI 1.07–1.77), and long-term mortality (HR: 1.37; 95% CI 1.27–1.49) [14]. POAF was more strongly associated with stroke in patients undergoing non-cardiac surgery than in those undergoing cardiac surgery (HR: 2.00; 95% CI 1.70–2.35 and HR: 1.20; 95% CI 1.07–1.34, respectively) [14], possibly because of a stronger reversible physical trigger from direct cardiac manipulation during cardiac surgery.

Currently, the use of oral anticoagulants (OACs) in POAF patients is still debated, and current practices are heterogeneous. Potential issues include the lack of robust evidence, perioperative bleeding risk, and the common perception that POAF is a transient condition [15–17]. The AF-SCREEN International Collaboration (http://www.afscreen.org/) developed an online anonymous survey to provide an overall picture of current practice on the detection and management of POAF occurring after CABG or non-cardiac surgery and potential issues concerning patient treatment.

## Methods

An online questionnaire consisting of 17 multiple choice or rank questions was developed by the AF-SCREEN International Collaboration, an international network of 170 key players (including patient advocates) in the field of AF from 37 countries, whose aim is to promote discussion and research about screening for unknown or under-treated AF, as a way to reduce stroke and death. The development of the questionnaire involved a collaborative effort and implied multiple rounds of revisions and refinements until full consensus among the group was achieved. Three key domains were identified as potential sources of bias and addressed accordingly: question design, questionnaire structure, and administration process. The questionnaire was distributed by e-mail to an international network of healthcare professionals working in the field of arrhythmias, stroke, cardiac surgery, and postoperative cardiac rehabilitation. The questionnaire was anonymous and complied with the European General Data Protection Regulation (Supplementary Appendix). No individual answer was mandatory to complete the survey. Therefore, missing data were excluded for the purpose of the present analysis (< 5%). Counts and percentage or weighted means are reported for each answer of the survey. Statistical analyses and charts were performed using R 4.2.2 for MacOS.

## Results

Between June 2023 and June 2024, a total of 158 participants completed the survey. Among them, 70 (44%) were AF-SCREEN members. The geographical region of the participants encompassed 25 countries, with the majority responding from Italy (30.4%) and Canada (27.2%) (Table 1). The age distribution of the respondents is detailed in Supplementary Table 2, with the majority aged between 51 and 65 years (33%). The predominant subspecialty was electrophysiology (43%), followed by general cardiology (28%), internal medicine (14%), neurology (4%), and other fields, including primary care and allied health professions (11%).

**Table 1:** Country of practice of the respondents

Nation	n	%
Austria	1	0.6
Belgium	1	0.6
Canada	43	27.2
Denmark	4	2.5
Finland	1	0.6
France	3	1.9
Germany	12	7.6
Greece	3	1.9
Ireland	1	0.6
Israel	4	2.5
Italy	48	30.4
Japan	3	1.9
Mexico	1	0.6
Netherlands	2	1.3
Norway	1	0.6
Republic of Korea	1	0.6
Romania	1	0.6
Saudi Arabia	1	0.6
Slovakia	1	0.6
Spain	1	0.6
Sweden	5	3.2
Thailand	1	0.6
UK	5	3.2
Uruguay	3	1.9
USA	11	7.0

### Detection and management of postoperative atrial fibrillation after coronary artery bypass grafting

**Fig. 1:**
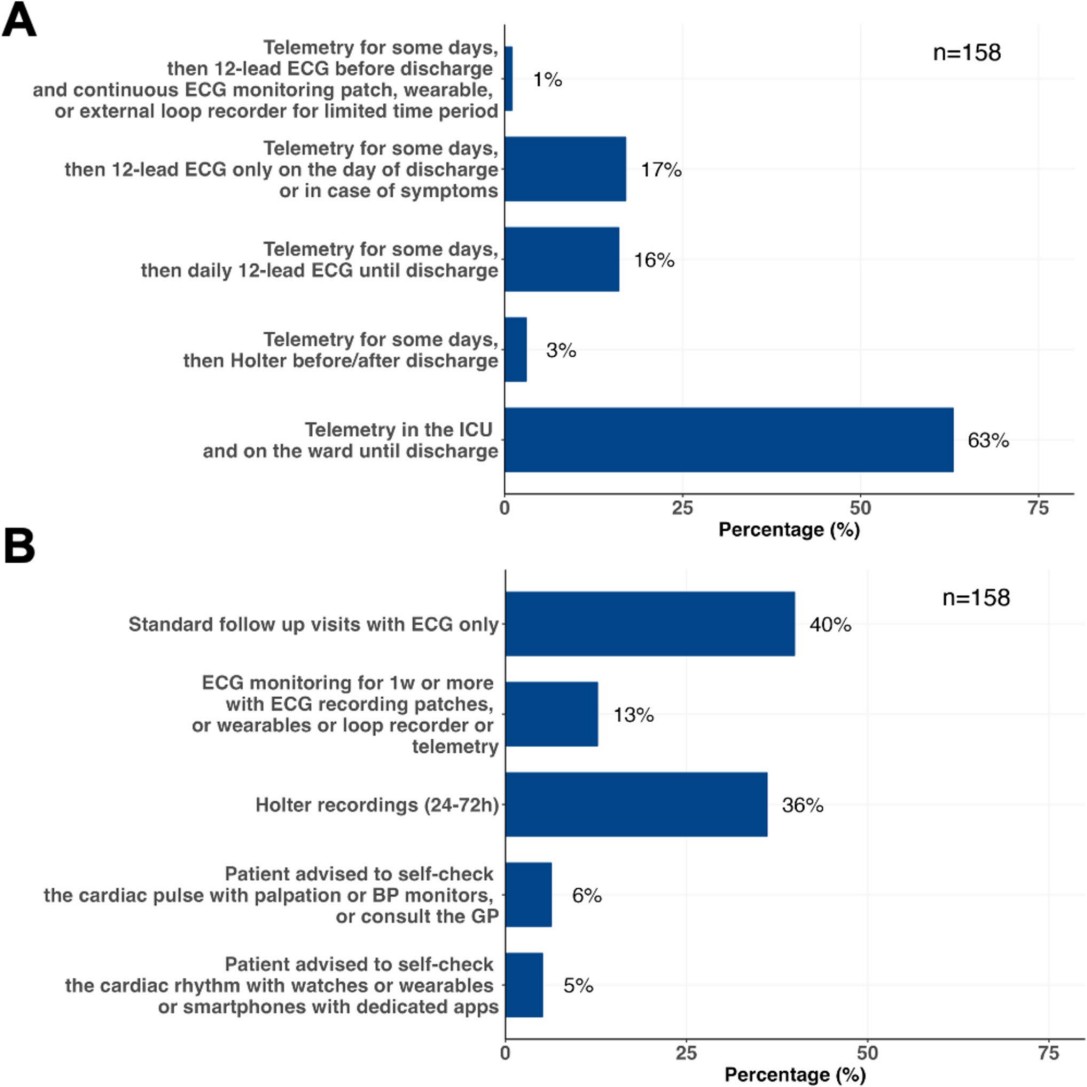
Panel A shows the methods that participants used in their hospitals to detect postoperative atrial fibrillation (POAF) after coronary artery bypass grafting (CABG). Panel B shows the reported methods for monitoring atrial fibrillation recurrences during follow-up in patients with POAF after CABG and subsequent resumption of sinus rhythm. *BP* blood pressure, *GP* general practitioner, *ICU* intensive care unit, *w* week

Participants were asked to report the methods used in their hospitals to detect POAF after CABG (Fig. 1A). The majority indicated that telemetry was employed both in the intensive care unit (ICU) and on the ward until discharge (63%). A subset of respondents (17%) reported using telemetry for some days, followed by 12-lead ECG only on the day of discharge or if symptoms occurred. 16% mentioned telemetry for some days, followed by daily 12-lead ECG until discharge and a small fraction reported to also add Holter-ECG before/after discharge (3%) or continuous ECG monitoring patch/ wearables/loop recorders for a limited time period (1%).

In case of POAF after CABG surgery with subsequent resumption of sinus rhythm, the greatest number of respondents (40%) reported that physicians in their hospital do not usually plan dedicated methods for monitoring AF recurrences during follow-up, relying solely on ECG at the time of cardiology visits. 36% of respondents usually plan 24–72 h Holter recordings, 13% plan ECG monitoring for 1 week or more with ECG patches or wearable devices or loop recorder, 6% advise patients to self-check the cardiac pulse (via palpation, blood pressure monitors or consultation with the primary care physician), and 5% advise patients to self-check the cardiac rhythm using wearable devices or smartphones with dedicated applications (app) (Fig. 1B).

**Fig. 2:**
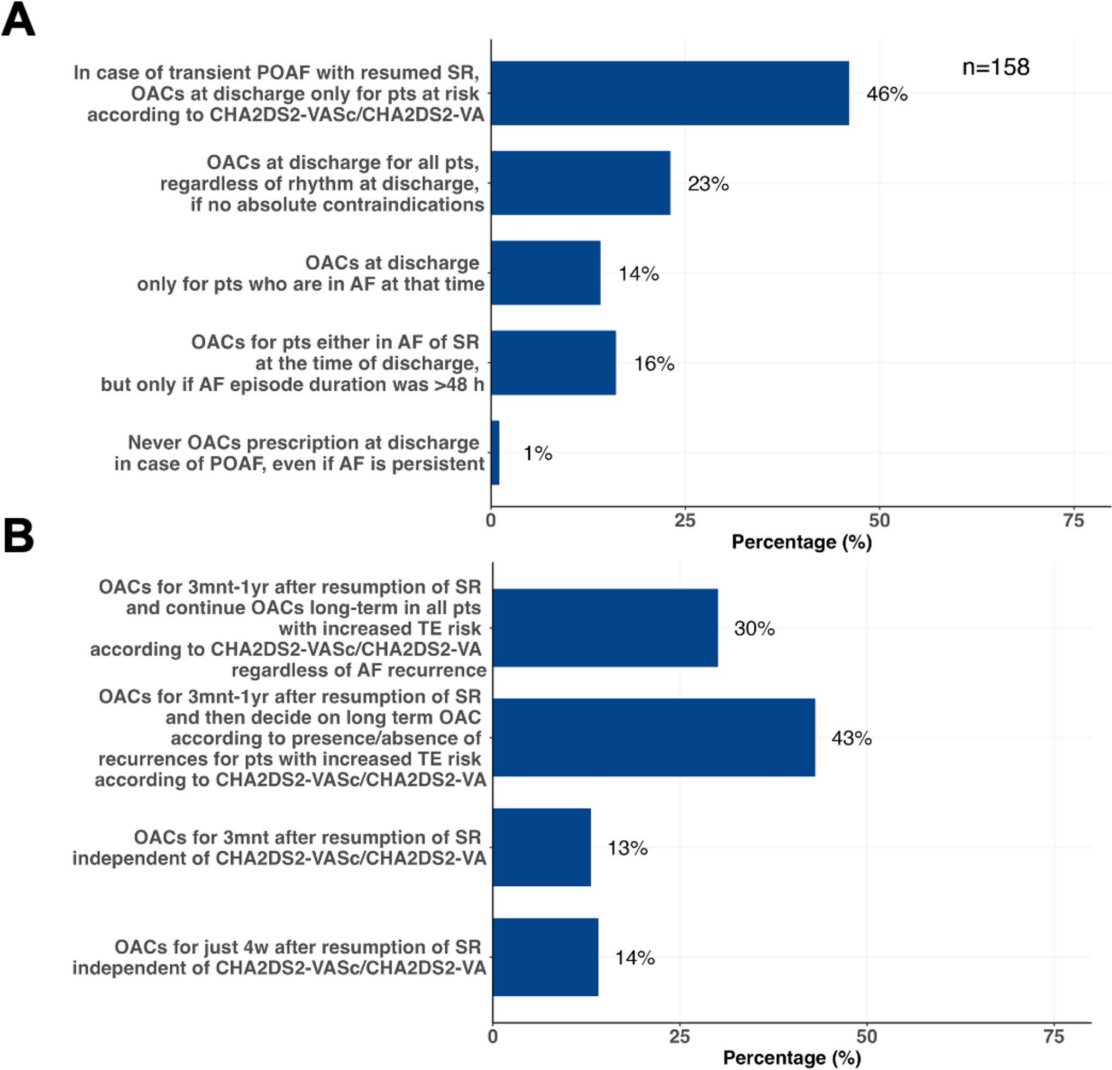
Panel A shows short-term oral anticoagulant prescription in the context of postoperative atrial fibrillation (POAF) after coronary artery bypass grafting (CABG). Panel B shows longer-term management. *AF* atrial fibrillation, *mnt* months, *OACs* oral anticoagulants, *POAF* postoperative atrial fibrillation, *pts* patients, *SR* sinus rhythm, *TE* thromboembolic, *w* weeks, *yrs* years

Participants were also asked to report how they managed OAC prescription in the context of POAF after CABG. The largest group (46%) reported that, for patients who experienced transient AF and resumed sinus rhythm, OACs are prescribed at discharge if patients are at risk according to CHA_2_DS_2_-VASc/CHA_2_DS_2_-VA scores. Another 23% of respondents consider prescribing OACs for all patients, regardless of rhythm at discharge, provided there are no absolute contraindications. 16% consider prescribing OACs for patients in either AF or sinus rhythm at the time of discharge, but only if AF episode duration exceeded 48 h, and 14% consider prescribing OACs only to patients who are in AF at the time of discharge (Fig. 2A).

In case of POAF with subsequent resumption of sinus rhythm, the largest proportion of respondents (43%) reported prescribing OACs for a duration of 3 months to 1 year, after long-term anticoagulation is decided according to presence or absence of recurrences for patients with increased thromboembolic risk according to CHA_2_DS_2_VASc/CHA2DS_2_VA scores. A slightly smaller group (30%) continue OACs long-term in all patients with increased thromboembolic risk regardless of AF recurrence, 14% prescribe OACs for just 4 weeks after resumption of sinus rhythm independent of CHA_2_DS_2_VASc/CHA_2_DS_2_VA scores, and 13% prescribe OACs for only 3 months (Fig. 2B).

The minimum reported duration of POAF episode considered to start OACs was: any duration of 30 s or more for 28% of respondents, followed by 6 h or more (22%), 24 h or more (19%), 6 min or more (14%), duration does not matter (11%), and lastly 48 h or more (6%) (Supplementary Fig. 1).

The main concern about long-term anticoagulation in patients with POAF after CABG and at risk of stroke according to CHA_2_DS_2_VASc/CHA_2_DS_2_VA score was lack of randomized controlled trials (RCTs) (weighted mean 1.42), followed by no clear evidence of net benefit from available observational studies (2.46), unclear guidelines (2.97), risk of bleeding (3.40), and potential lack of patient adherence to OACs (3.74) (Supplementary Fig. 2).

Regarding cardiologist involvement in decision-making for OAC prescriptions and discharge/follow-up plans for patients with POAF after CABG, 35% of respondents reported that cardiologists are involved in most cases, 29% said they are involved sometimes, 21% said they are always involved, 12% said rarely, and 3% said never (Supplementary Fig. 3).

### Detection and management of postoperative atrial fibrillation in the setting of non-cardiac surgery

**Fig. 3:**
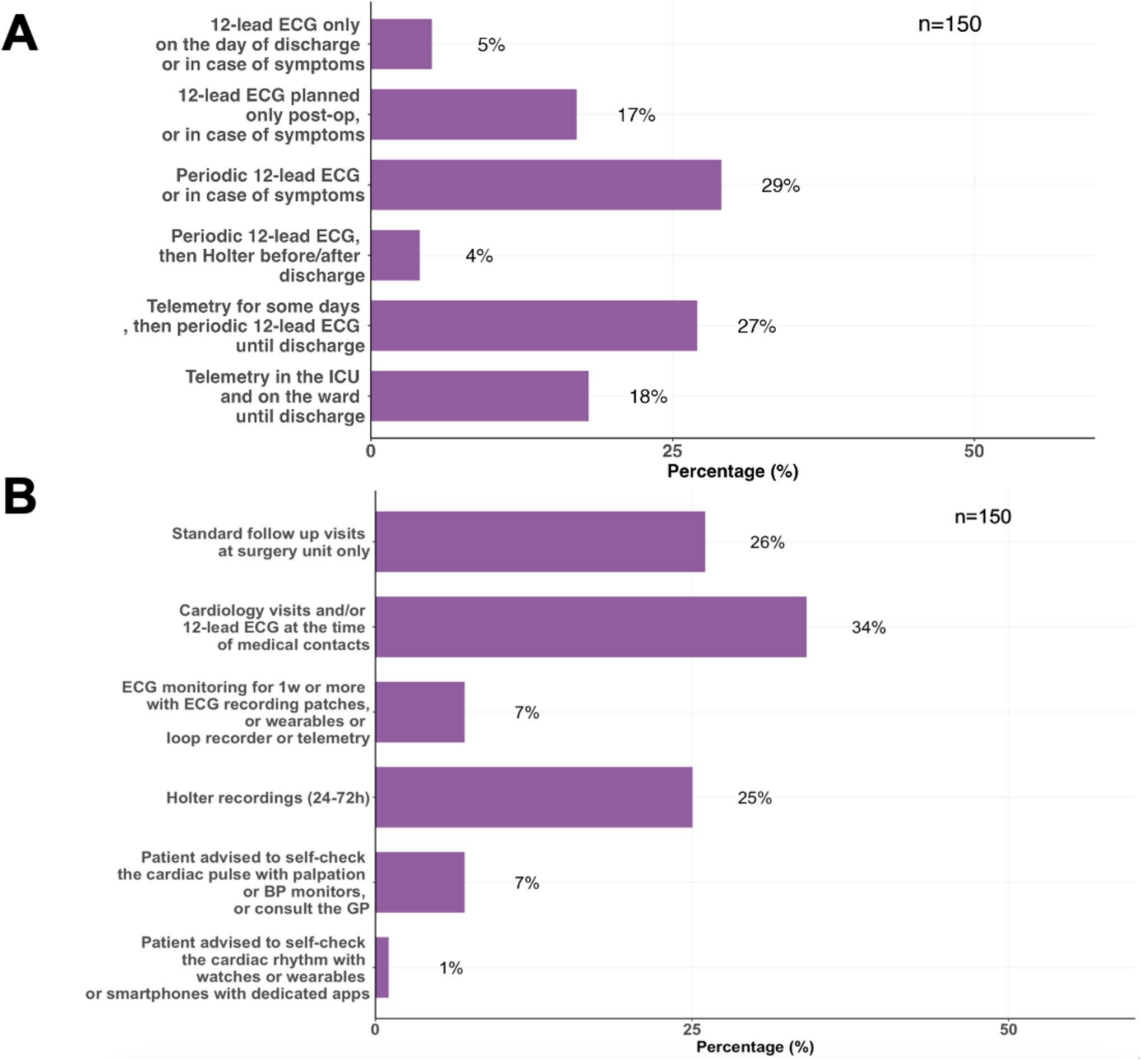
Panel A shows the methods that participants used in their hospitals to detect postoperative atrial fibrillation (POAF) after non-cardiac surgery. Panel B shows the reported methods for monitoring atrial fibrillation recurrences during follow-up in patients with POAF after non-cardiac surgery and subsequent resumption of sinus rhythm. *BP* blood pressure, *GP* general practitioner, *ICU* intensive care unit, *post-op* postoperatively, *w* week

In the setting of non-cardiac surgery, the methods reported for POAF detection were as follows: periodic 12-lead ECG or symptoms-activated ECG (29%), telemetry for some days, followed by periodic 12-lead ECGs until discharge (27%), telemetry in ICU an on the ward until discharge (18%), 12-lead ECG only in case of symptoms (17%), 12-lead ECG only on the day of discharge or in case of symptoms (5%), and periodic 12-lead ECG followed by Holter before or after discharge (4%) (Fig. 3A).

With regard to AF recurrence monitoring in patients with transient POAF and subsequent resumption of sinus rhythm, the largest group (34%) reported to plan cardiology visits with 12-lead ECGs. A slightly smaller group plan (26%) only normal follow-up visits at the surgery unit, with 12-lead ECG according to physician discretion and a similar proportion (25%) plan 24–72 Holter recordings. 7% plan ECG monitoring for 1 week or more with ECG patches or wearable devices or loop recorder, 7% advise patients to self-check the cardiac pulse (via palpation, blood pressure monitors or consultation with the primary care physician), and 1% advise patients to self-check the cardiac rhythm using wearable devices or smartphones with dedicated apps (Fig. 3B).

**Fig. 4:**
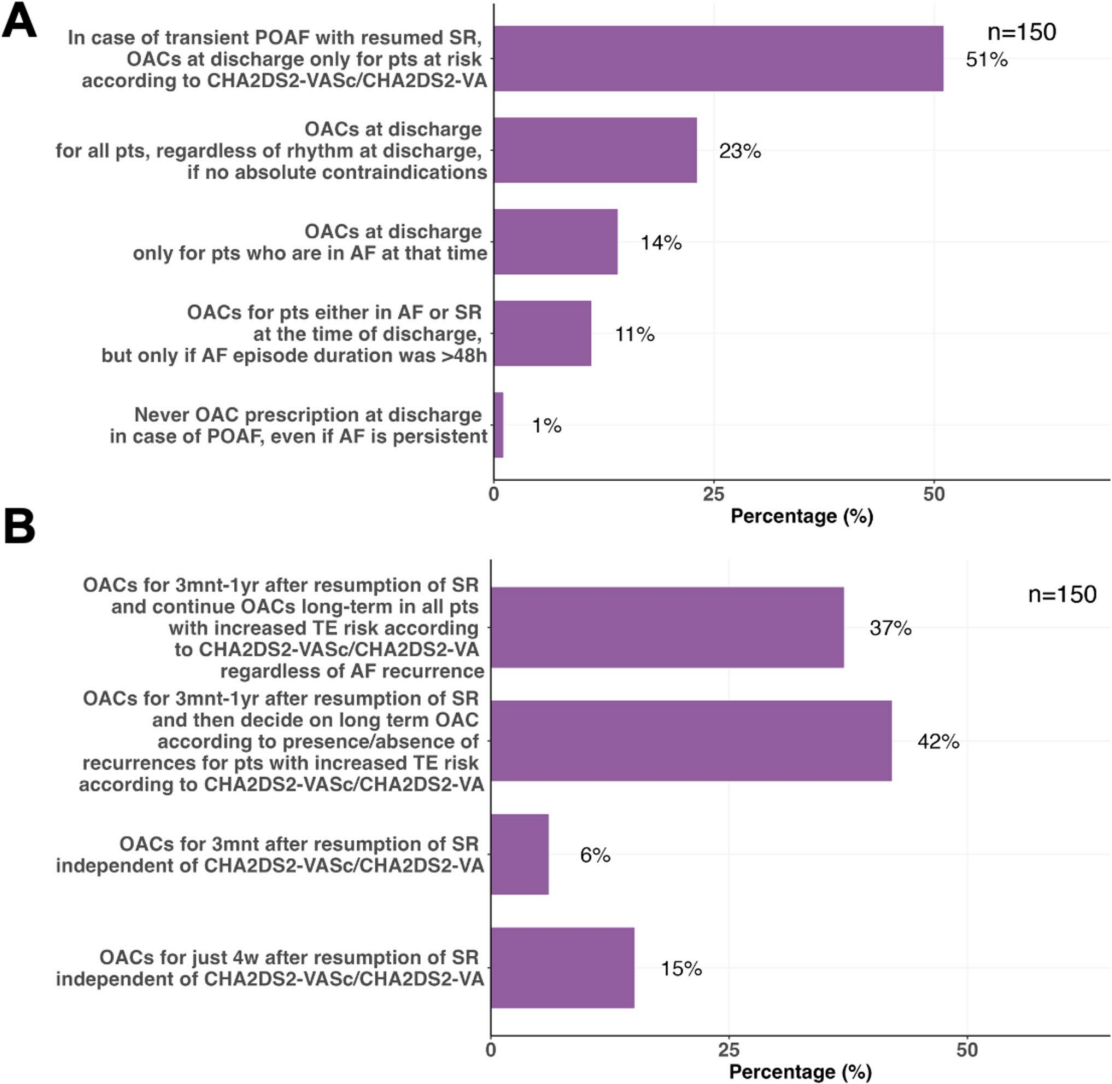
Panel A shows short-term oral anticoagulant prescription in the context of postoperative atrial fibrillation (POAF) after non-cardiac surgery. Panel B shows longer term management. *AF* atrial fibrillation, *mnt* months, *OACs* oral anticoagulants, *POAF* postoperative atrial fibrillation, *pts* patients, *SR* sinus rhythm, *TE* thromboembolic, *w* weeks, *yrs* years

Regarding OAC prescription in relation to the heart rhythm at discharge, the majority (51%) of respondents reported that, for patients who experienced transient AF and resumed sinus rhythm, OACs are prescribed at discharge if patients are at risk according to CHA_2_DS_2_VASc/CHA_2_DS_2_VA scores. 23% reported to consider prescribing OACs for all patients, regardless of rhythm at discharge, provided there are no absolute contraindications. 14% consider prescribing OACs only to patients who are in AF at the time of discharge, and 11% consider prescribing OACs for patients in either AF or sinus rhythm at the time of discharge, but only if AF episode duration exceeded 48 h, and 1% never prescribe OACs at discharge in case of POAF, even if AF is persistent (Fig. 4A).

OACs in patients resuming sinus rhythm were reported to be prescribed for 3 months to 1 year after resumption of sinus rhythm, followed by a decision on long-term anticoagulation according to presence or absence of recurrences for patients with increased thromboembolic risk according to CHA_2_DS_2_VASc/CHA_2_DS_2_VA scores by 42% of respondents. 37% of respondents reported to continue OACs long-term in all patients with increased thromboembolic risk regardless of AF recurrence, 15% to prescribe OACs for just 4 weeks after resumption of sinus rhythm independent of CHA_2_DS_2_-VASc/CHA_2_DS_2_-VA scores, and 6% prescribe OACs for only 3 months (Fig. 4B).

The minimum reported duration of POAF episode after non-cardiac surgery considered to start OACs was: any duration of 30 s or more for 33% of respondents, followed by 6 h or more (24%), 24 h or more (14%), 6 min or more (11%), duration does not matter (11%), and lastly 48 h or more (7%) (Supplementary Fig. 4).

The main concerns about long-term anticoagulation in patients with POAF after non-cardiac surgery were as follows: lack of RCTs, no clear evidence of net benefit from available observational studies, unclear guidelines, risk of bleeding, and potential lack of patients’ adherence to OACs (weighted mean 1.49, 2.46, 3.06, 3.29, and 3.69, respectively) (Supplementary Fig. 5).

Finally, regarding cardiologist involvement in decision-making for OAC prescriptions and discharge/follow-up plans for patients with POAF after non-cardiac surgery, 47% of respondents reported that cardiologists are involved in most cases, 27% said they are involved sometimes, 19% said they are always involved, 6% said rarely, and 1% said never (Supplementary Fig. 6).

## Discussion

In the present survey, we explored current practices for detecting and managing POAF in the context of both CABG and non-cardiac surgery. The main findings were as follows: (i) the most commonly reported methods for POAF detection after CABG and after non-cardiac surgery were telemetry until discharge and periodic 12-lead ECGs, respectively; (ii) in both contexts, most respondents reported that no structured follow-up for monitoring AF recurrences during follow-up was planned/only ECG at the time of cardiology visit and the role of wearable devices is marginal. (iii) There was substantial heterogeneity in terms of OAC prescription and treatment duration, and the most commonly reported barrier was the lack of randomized trial data. Finally, (iv) only approximately one third—one half of respondents reported that cardiologists are involved in most cases in decision-making for OACs prescription and discharge/follow-up plan.

### POAF after cardiac surgery

Our survey highlights that there is substantial heterogeneity in the reported methods for POAF detection and that they differ between the CABG and non-cardiac surgery settings. In the former, the preferred method for POAF detection was telemetry on the ward until discharge.

Documentation of AF recurrence following hospital discharge is the simplest method of risk stratifying patients with POAF following surgery. A meta-analysis of eight studies showed that the incidence rate of POAF recurrence identified through intermittent non-invasive monitoring in the first 4 weeks post-discharge was 28.3%, while the incidence rate identified through prolonged implanted continuous monitoring was 61–100% within 2 years [5]. Another meta-analysis aiming to estimate the rate of AF recurrence over the long-term in patients experiencing POAF within 30 days after cardiac surgery showed that the pooled AF recurrence rates (detected by an implantable loop recorder) were 17.8% (95% CI 11.9%–23.2%) at 3 months, climbing to 35.3% (27.6%–42.2%) at 18 months [18]. For the purpose of AF recurrences detection, the majority of respondents reported no structured follow-up or 12-lead ECG only at the time of outpatient visits. Long-term monitoring strategies (e.g.: 1–2 weeks Holter, patches, loop recorder) and the use of wearable digital devices were not commonly adopted despite it is known that shorter monitoring intensities (24–/48-h Holter) are less sensitive for detecting AF recurrence [19]. This finding may reflect an underestimation by physicians of the risk of disease progression and of the negative impact of POAF on patient prognosis. In addition, it may partially be explained by a lack of resources and personnel in some centers to manage the increased workload posed by remote monitoring of patients [20, 21]. The implementation of effective monitoring strategies may help to differentiate between transient, reversible POAF and non-surgical AF, and thus to better identify those patients that may benefit the most of disease-specific therapies.

The use of long-term OACs in POAF patients remains debated. The 2020 edition of the European Society of Cardiology (ESC) guidelines [15] recommend long-term OACs in patients at risk for stroke, considering the anticipated net clinical benefit of OAC therapy and informed patient preferences, with a class IIb, level of evidence (LOE) B for cardiac surgery patients. This recommendation slightly changed in the 2024 edition [22] (Class IIa, LOE B), highlighting this knowledge gap has not yet been filled. The American College of Cardiology (ACC)/American Heart Association (AHA) 2023 guidelines [16] state that it is reasonable to administer OACs for 60 days after cardiac surgery and to reevaluate the need for longer-term therapy at that time (Class IIa, LOE B-NR).

Our survey highlights substantial heterogeneity in the reported indication for OACs prescription in POAF patients. The CHA_2_DS_2_-VASc/CHA_2_DS_2_-VA score, presence of AF at discharge and AF duration > 48 h emerged as important factors influencing the decision. Interestingly, the presence of AF recurrences was reported as an important determinant for a decision on long-term anticoagulation, but a considerable proportion of respondents did not prescribe long-term OAC, irrespective of cardiac rhythm and CHA_2_DS_2_-VASc/CHA_2_DS_2_-VA score. There was also disagreement around the minimum duration of POAF episode considered to start OACs, ranging between 30 s and more than 48 h. Of note, clinical AF detected by surface ECG has different prognostic implications as compared to atrial high-rate episodes (AHRE) [23–25]. Therefore, the monitoring method used to detect AF has substantial implications. Nonetheless, a recent study on 1,031 patients (43% developing POAF; mean follow-up of 4.7 ± 2.4 years) showed that late AF was significantly more likely among patients with POAF than those without (23% vs 6%; P < 0.001), with the highest incidence (38%) in those with POAF duration > 48 h [26].

The most commonly reported concern related to OAC prescription was the lack of relevant RCTs. Only approximately one third of respondents reported that cardiologists were involved in decision-making for OACs prescription and planning discharge and follow-up.

Although our survey focused primarily on POAF detection and management in CABG patients, some findings may extend to other types of cardiac surgery, such as valve or aortic procedures, as these are often performed by the same surgical teams in similar healthcare settings. Perioperative and postoperative monitoring strategies, as well as protocols for OAC prescription, are likely to overlap due to shared resources and infrastructure. However, differences in patient profiles and specific surgical risks may limit the generalizability of our results, particularly regarding the indications for long-term OAC use.

### POAF after non-cardiac surgery

The preferred method for POAF detection after non-cardiac surgery was reported to be periodic ECGs or ECG performed in case of symptoms. Only approximately one fifth of the respondents reported that telemetry was the most commonly adopted method. This difference, as compared to the setting of POAF occurring after cardiac surgery, may be in part attributed to the substantial differences in the organization and structure of cardiac surgery as compared to non-cardiac surgery units and also by the lower incidence of the arrhythmia, which might also be underestimated by the treating physicians. The reported prevalence of POAF is highly variable, ranging from 0.5% to 15% in non-thoracic, non-cardiac surgery, up to 20% after non-cardiac thoracic surgery [2, 27]. Of note, a recent meta-analysis on 3,718,587 patients showed a fourfold higher risk of stroke associated with POAF after non-cardiac surgery (OR 4.05; 95% CI 2.91–5.62), suggesting that in this setting, AF may be not only be the result of an external, transient trigger, but also an expression of an atrial substrate favorable for the subsequent development of AF [11].

In our survey, the majority of respondents reported no structured follow-up or 12-lead ECG only at the time of outpatient visits as the preferred method for AF recurrences detection. Considering the non-negligible rate of arrhythmia recurrence, a more adequate surveillance should be planned, especially in patients with risk factors for AF recurrence [28].

We observed a substantial variability among anticoagulation practices following POAF occurring after non-cardiac surgery, and the reported likelihood of OAC prescription did not substantially change as compared with POAF occurring after CABG. Current and former ESC guidelines and ACC/AHA guidelines recommend OAC prescription with a class IIa, LOE B [15, 16, 22] in the setting of non-cardiac surgery. These findings suggest the need for more studies addressing this topic. In fact, the most commonly reported concern on OAC prescription in our survey was the lack of RCT data. Geographic and institutional factors may also contribute to the observed variability in POAF management, influenced by differences in healthcare systems, resource availability, and institutional protocols. ESC 2024 guidelines recommend long-term OAC use in patients with POAF after cardiac and non-cardiac surgery at elevated thromboembolic risk to prevent ischemic stroke and thromboembolism [15]. On the other hand, ACC/AHA 2023 guidelines recommend a structured approach to OAC therapy after cardiac surgery, with a Class IIa, LOE B-NR recommendation for a 60-day course of OAC followed by reevaluation. In patients with AF who are identified in the setting of non-cardiac surgery, outpatient follow-up for thromboembolic risk stratification and decision-making on OAC initiation or continuation is suggested (Class IIa; LOE B-NR) [16]. Similarly, the Asia–Pacific Heart Rhythm Society (APHRS) guidelines emphasize the importance of individualized anticoagulation decisions in POAF patients, considering patient-specific stroke and bleeding risks, but lack strong evidence-based recommendations for POAF-specific scenarios.

Finally, our survey highlighted that cardiologists were routinely involved in decision-making on OAC prescription in the setting of POAF occurring after non-cardiac surgery in only less than half of the cases.

Our survey results reinforce the need for a more standardized approach to arrhythmia surveillance, identification of better predictors of AF recurrences and stroke, perioperative treatment strategies [29, 30], and implementation of multidisciplinary teams.

Our survey underscores the urgent need for well-designed, targeted RCTs. The lack of such trials represents a critical barrier to standardizing POAF detection and management. Furthermore, the absence of RCTs, widely regarded as the cornerstone of evidence-based medicine, combined with inconclusive findings from observational studies, contributes to ambiguity in guidelines and challenges clinical decision-making. Notably, large multinational RCTs of OAC are ongoing in both populations. For POAF following CABG, the PACES trial (ClinicalTrials.gov Identifier: NCT04045665) and for POAF following non-cardiac surgery, ASPIRE-AF (NCT03968393—https://clinicaltrials.gov/study/NCT03968393) are underway and may provide further guidance on requirement for OAC therapy.

This survey does not address the role of antiarrhythmic drug (AAD) prescriptions at discharge, which remains an unresolved issue. Although the benefits of perioperative amiodarone therapy are well-established [15, 16], the optimal long-term management strategy in this context is still unclear. Recent studies showed the advantages of early rhythm control over rate control [31], but postoperative patients have not been thoroughly studied. These patients exhibit unique characteristics, including specific patient profiles and AF traits, which complicate the choice between rate and rhythm control [32, 33]. Consequently, the risk–benefit ratio of AADs or catheter ablation requires more careful consideration in this population.

### Limitations

The main limitations of the present survey are potential selection bias and inaccuracy of self-reported data.

Subjects participating in the survey might be more interested in this topic, and thus represent a sample of relatively more expert health care providers. However, even in this most engaged group, there is substantial practice variation and uncertainty in best evidence. Considering the administration process of the present survey, it was not possible to calculate the participants’ compliance rate. Finally, the relatively small sample size of this survey, along with the subspecialty filed of the respondents, most of whom were neither surgeons nor postoperative intensive care specialists, may limit the generalizability of the results.

## Conclusion

For both CABG and non-cardiac surgery, the reported methods for POAF detection and recurrence monitoring were heterogeneous, and prescription patterns for OACs varied greatly. The most frequently reported concern about long-term anticoagulation was the lack of randomized data, highlighting the urgent need for well-designed studies to inform daily clinical practice. By identifying critical gaps in the standardization of POAF management, our survey provides a foundation for future prospective studies, especially RCTs, aimed at addressing these uncertainties.

## Supplementary Information

Below is the link to the electronic supplementary material.

Supplementary file1 (DOCX 1021 KB)

## Data Availability

Data will be shared upon reasonable request to the corresponding author.
