# Supplementary material for: Detection and management of postoperative atrial fibrillation after coronary artery bypass grafting or non-cardiac surgery: a survey by the AF-SCREEN International Collaboration
Source: Intern Emerg Med. 2025 Feb 8;20(3):739–49. doi: 10.1007/s11739-025-03861-2 (PMC12009235; doi:10.1007/s11739-025-03861-2)
Supplement: Supplementary file 1 — Supplementary file1 (DOCX 1021 KB) [file 11739_2025_3861_MOESM1_ESM.docx]

**SUPPLEMENTARY MATERIAL**

**INDEX**

**Supplementary Appendix.** Online Questionnaire.

**Supplementary Table 1.** Age distribution of the respondents.

**Supplementary Figure 1.** Minimum duration of postoperative atrial fibrillation (POAF) episode required to consider oral anticoagulants prescription, in the setting of POAF after coronary artery bypass grafting.

**Supplementary Figure 2.** Main issues concerning decisions about long term anticoagulation in patients with postoperative atrial fibrillation in the setting of coronary artery bypass grafting in patients at increased stroke risk according to CHA_2_DS_2_-VASc/CHA_2_DS_2_-VA.

**Supplementary Figure 3.** Involvement of cardiologists in decision making for oral anticoagulants prescription and discharge/follow-up plan in the setting of postoperative atrial fibrillation occurring after coronary artery bypass grafting.

**Supplementary Figure 4.** Minimum duration of postoperative atrial fibrillation (POAF) episode required to consider oral anticoagulants prescription, in the setting of POAF after non-cardiac surgery.

**Supplementary Figure 5.** Main issues concerning decisions about long term anticoagulation in patients with postoperative atrial fibrillation in the setting of non-cardiac surgery in patients at increased stroke risk according to CHA_2_DS_2_-VASc/CHA_2_DS_2_-VA.

**Supplementary Figure 6.** Involvement of cardiologists in decision making for oral anticoagulants prescription and discharge/follow-up plan in the setting of postoperative atrial fibrillation occurring after non-cardiac surgery.

**Supplementary Appendix.** Online Questionnaire.

Q1. In which country do you work? ________________________________

Q2. Are you an AF-SCREEN Member?

1. Yes
2. No

Q3. What is your field? (one choice)

1. General cardiologist
2. Internal medicine physician
3. Electrophysiologist
4. Neurologist/stroke physician
5. Primary care physician/general practitioner
6. Cardiac surgeon
7. Thoracis/vascular surgeon
8. Other surgeon
9. Nurse/allied health professional
10. Non clinical or not a health professional
11. Other

**POAF after CABG surgery [Excluding cases with associated valve surgery] (Q 4-Q10)**

Q4. For detecting POAF after CABG surgery what methods are used in your hospital?

1. Telemetry in the intensive care unit and in the ward until discharge

2. Telemetry for some days, then daily 12-lead ECG until discharge

3. Telemetry for some days, then 12-lead ECG only on the day of discharge or in case of symptoms

4. Telemetry for some days, then Holter before/after discharge

5. Telemetry for some days , then 12-lead ECG before discharge and continuous ECG monitoring patch, wearable, or external loop recorder for limited time period

6. _ Telemetry for some days , then 12-lead ECG before discharge and continuous ECG monitoring by implanted loop recorder

Q5. For POAF in patients at risk according to CHA2DS2VASc/CHA2DS2VA occurring following CABG surgery, do you differentiate patients discharged in sinus rhythm (“transient AF”) and patients who are still in AF at discharge for a decision of prescription of anticoagulants?

1. I consider prescription of anticoagulants at discharge only in pts who are in AF at the time of discharge
2. I consider prescription of anticoagulants at discharge for all patients, regardless of rhythm at discharge, if no absolute contraindications
3. I consider prescription of anticoagulants both for patients who are in AF at the time of discharge, and also those with resumed sinus rhythm, but only if AF episode duration was >48 h
4. For patients who had transient AF and resumed sinus rhythm, I prescribe anticoagulants at discharge only if they are at risk according to CHA2DS2VASc/CHA2DS2VA
5. I never prescribe anticoagulants at discharge in case of POAF even if persistent

Q6. In case of POAF occurring in the setting of CABG surgery, with subsequent resumption of sinus rhythm, for how long do you advice use of anticoagulants ?

1. I prescribe anticoagulants just for 4 weeks after resumption of sinus rhythm, independent of CHA2DS2VASc/CHA2DS2VA score
2. I prescribe anticoagulants for 3 months after resumption of sinus rhythm, independent of CHA2DS2VASc/CHA2DS2VA
3. I prescribe anticoagulants for 3 months - 1 year after resumption of sinus rhythm, and then decide on long term anticoagulation according to presence/absence of recurrences for pts with increased thromboembolic risk according to CHA2DS2VASc/CHA2DS2VA
4. I prescribe anticoagulants for 3 months - 1 year after resumption of sinus rhythm and continue anticoagulants long-term in all patients with increased thromboembolic risk according to CHA2DS2VASc/CHA2DS2VA regardless of AF recurrence

Q7. In your hospital are cardiologists involved in decision making for anticoagulant prescription and discharge/follow up plan for patients who had POAF occurring in the setting of CABG surgery ?

1. Yes, always

2. Yes, in most cases

3. Only sometimes

4. Rarely

5. Never

Q8. In case of POAF in the setting of CABG surgery for what minimum duration of POAF episode would you consider anticoagulation?

1. for any duration of POAF > 30 s

2. for POAF episodes lasting 6 min or more

3. for POAF episodes lasting 6 hours or more

4. for POAF episodes lasting 24 hours or more

5. for POAF episodes lasting 48 hours or more

6. Duration of POAF does not matter

Q9. In your view what are the main issues concerning decisions about long term anticoagulation in patients with POAF in the setting of CABG surgery in patients with increased stroke risk according to CHA2DS2VASc/CHA2DS2VA ? ( please rank according to 1= most important, 5= least important)

- Lack of randomized controlled studies ______
- No clear evidence of net benefit from available observational studies ______
- Unclear guidelines ______
- Risk of bleeding ______
- Patients adherence to oral anticoagulant ______

Q10. In case of POAF after CABG surgery with subsequent resumption of sinus rhythm, do physicians in your hospital usually plan dedicated methods for monitoring AF recurrences during follow up?

1. No, normal follow up visits with ECG at the time of cardiology visits
2. Yes, the patient is advised to self-check the cardiac pulse with palpation or blood pressure monitors, or to get primary care physician to check
3. Yes, the patient is advised to self-check the cardiac rhythm with watches or wearables or smartphones with dedicated apps
4. Yes, Holter recordings (24-72 hours) are usually planned
5. Yes, ECG monitoring for 1 week or more with ECG recording patches, or wearables or external loop recorder or telemetry is usually planned
6. Yes, use of implanted loop recorders is usually planned

**POAF in setting of NON-cardiac surgery. (Q11 – Q18)**

Q11. For detecting POAF after non-cardiac surgery what methods are used in your hospital ?

1. Telemetry in the intensive care unit and in the ward till discharge

2. Telemetry for some days post-op, then periodic 12-lead ECG till discharge

3. Periodic 12-lead ECG or in case of symptoms

3. 12-lead ECG only on the day of discharge or in case of symptoms

4. Periodic 12-lead ECG , then Holter before/after discharge

5. No 12-lead ECG planned post-op, unless symptoms

Q12. In case of POAF after non-cardiac surgery, with subsequent resumption of sinus rhythm, do physicians in your hospital usually plan dedicated methods for monitoring AF recurrences during follow up?

1. No, normal follow up visits are planned
2. Yes cardiology visits and/or 12-lead ECG at the time of medical visits are planned
3. Yes, the patient is advised to self-check the cardiac pulse with palpation or blood pressure monitors, or to get primary care physician to check
4. Yes, the patient is advised to self-check the cardiac rhythm with watches or wearables or smartphones with dedicated apps
5. Yes, Holter recordings (24-72 hours) are usually planned
6. Yes ECG monitoring for 1 week or more with ECG recording patches, or wearables or external loop recorder or telemetry is usually planned
7. Yes, use of implanted loop recorders is usually planned

Q13. In case of POAF occurring in the setting of non-cardiac surgery, do you differentiate patients discharged in sinus rhythm (“transient AF”) and patients who are still in AF at discharge for a decision of prescription of anticoagulants?

1. I consider prescription of anticoagulants at discharge only in pts who are in AF at the time of discharge
2. I consider prescription of anticoagulants at discharge for all patients, regardless of rhythm at discharge, if no absolute contraindications
3. I consider prescription of anticoagulants both for patients who are in AF at the time of discharge, and also those with resumed sinus rhythm, but only if AF episode duration was >48 h
4. For patients who had transient AF and resumed sinus rhythm, I prescribe anticoagulants at discharge only if they are at risk according to CHA2DS2VASc/CHA2DS2VA
5. I never prescribe anticoagulants at discharge in case of POAF even if persistent

Q14. In case of POAF occurring in the setting of non-cardiac surgery, with subsequent resumption of sinus rhythm, for how long do you advice use of anticoagulants ?

1. I prescribe anticoagulants just for 4 weeks after resumption of sinus rhythm, independent of CHA2DS2VASc/CHA2DS2VA score
2. I prescribe anticoagulants for 3 months after resumption of sinus rhythm, independent of CHA2DS2VASc/CHA2DS2VA
3. I prescribe anticoagulants for 3 months - 1 year after resumption of sinus rhythm, and then decide on long term anticoagulation according to presence/absence of recurrences for pts with increased thromboembolic risk according to CHA2DS2VASc/CHA2DS2VA
4. I prescribe anticoagulants for 3 months - 1 year after resumption of sinus rhythm and continue anticoagulants long-term in all patients with increased thromboembolic risk according to CHA2DS2VASc/CHA2DS2VA regardless of AF recurrence

Q15. In your hospital are cardiologists involved in decision making and discharge/follow up plan for patients who had POAF occurring in the setting of non-cardiac surgery ?

1. Yes, always

2. Yes, in most cases

3. Only sometime

4. Rarely

5. Never

Q16. In case of POAF in the setting of non-cardiac surgery for what duration of POAF episode would you consider anticoagulation?

1. for any duration of POAF > 30 s

2. for POAF episodes lasting 6 min or more

3. for POAF episodes lasting 6 hours or more

4. for POAF episodes lasting 24 hours or more

5. for POAF episodes lasting 48 hours or more

6. Duration of POAF does not matter

Q17. In your view what are the main problems in considering anticoagulation at long term in patients with POAF after non-cardiac surgery who have a CHA2DS2VASc/CHA2DS2VA scores indicating a risk of stroke/thromboembolism ? ( please indicate: 1= most important, 5= least important)

1. Lack of randomized controlled studies ______
2. No clear evidence of net benefit from available studies ______
3. Unclear guidelines ______
4. Risk of bleeding ______
5. Patients’ adherence ______

**Supplementary Table 1.** Age distribution of the respondents.

| **Age** | **n** | **%** |
| --- | --- | --- |
| 25-30 years-old | 15 | 9 |
| 31-40 years-old | 30 | 19 |
| 41-50 years-old | 47 | 30 |
| 51-65 years-old | 52 | 33 |
| >65 years-old | 14 | 9 |

**Supplementary Figure 1.** Minimum duration of postoperative atrial fibrillation (POAF) episode required to consider oral anticoagulants prescription, in the setting of POAF after coronary artery bypass grafting.

H, hours; min, minutes; POAF, postoperative atrial fibrillation; s, seconds.

**
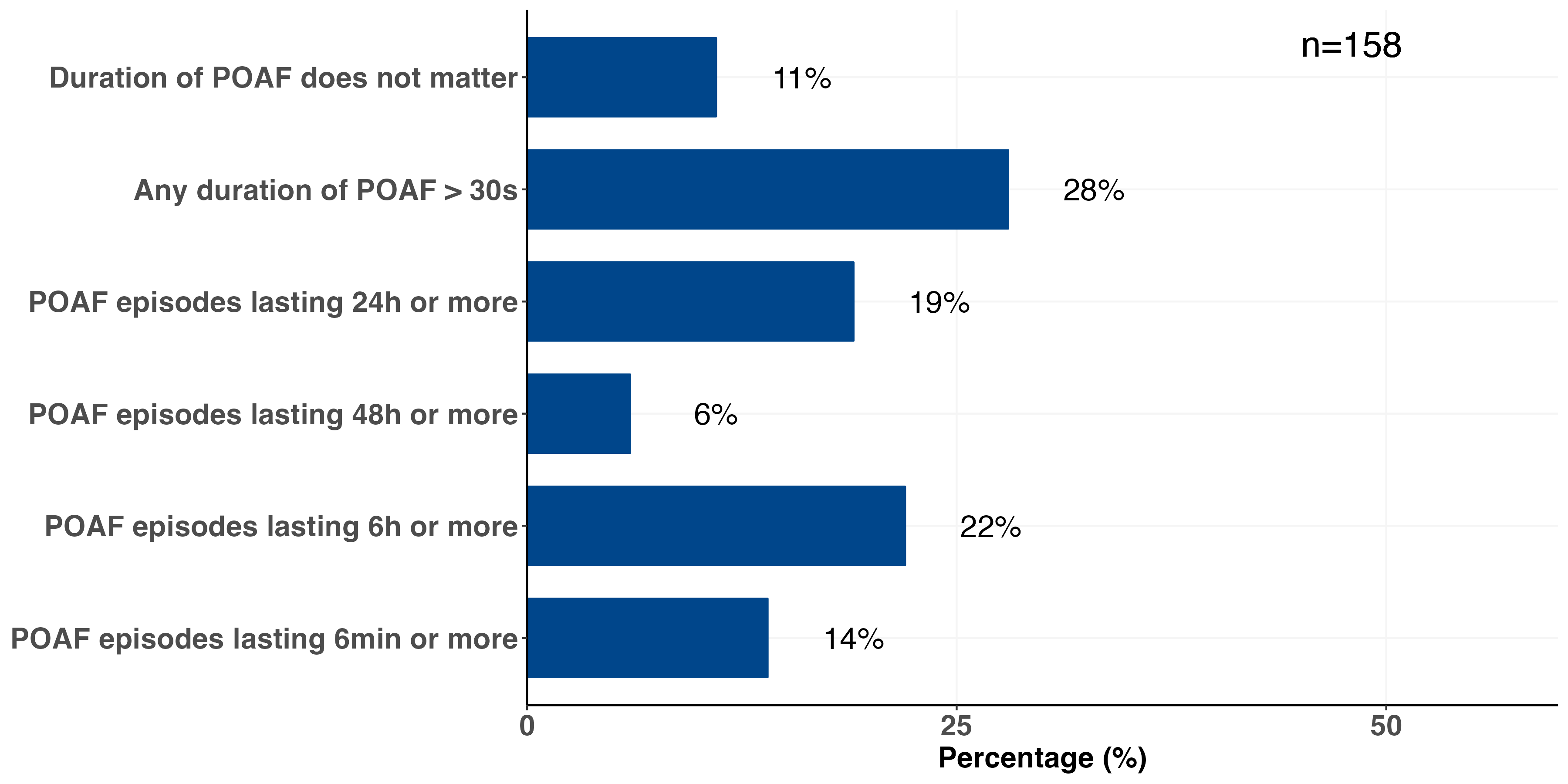
**

**Supplementary Figure 2.** Main issues concerning decisions about long term anticoagulation in patients with postoperative atrial fibrillation in the setting of coronary artery bypass grafting in patients at increased stroke risk according to CHA_2_DS_2_-VASc/CHA_2_DS_2_-VA.

OACs, oral anticoagulants; RCTs, randomized controlled trials.


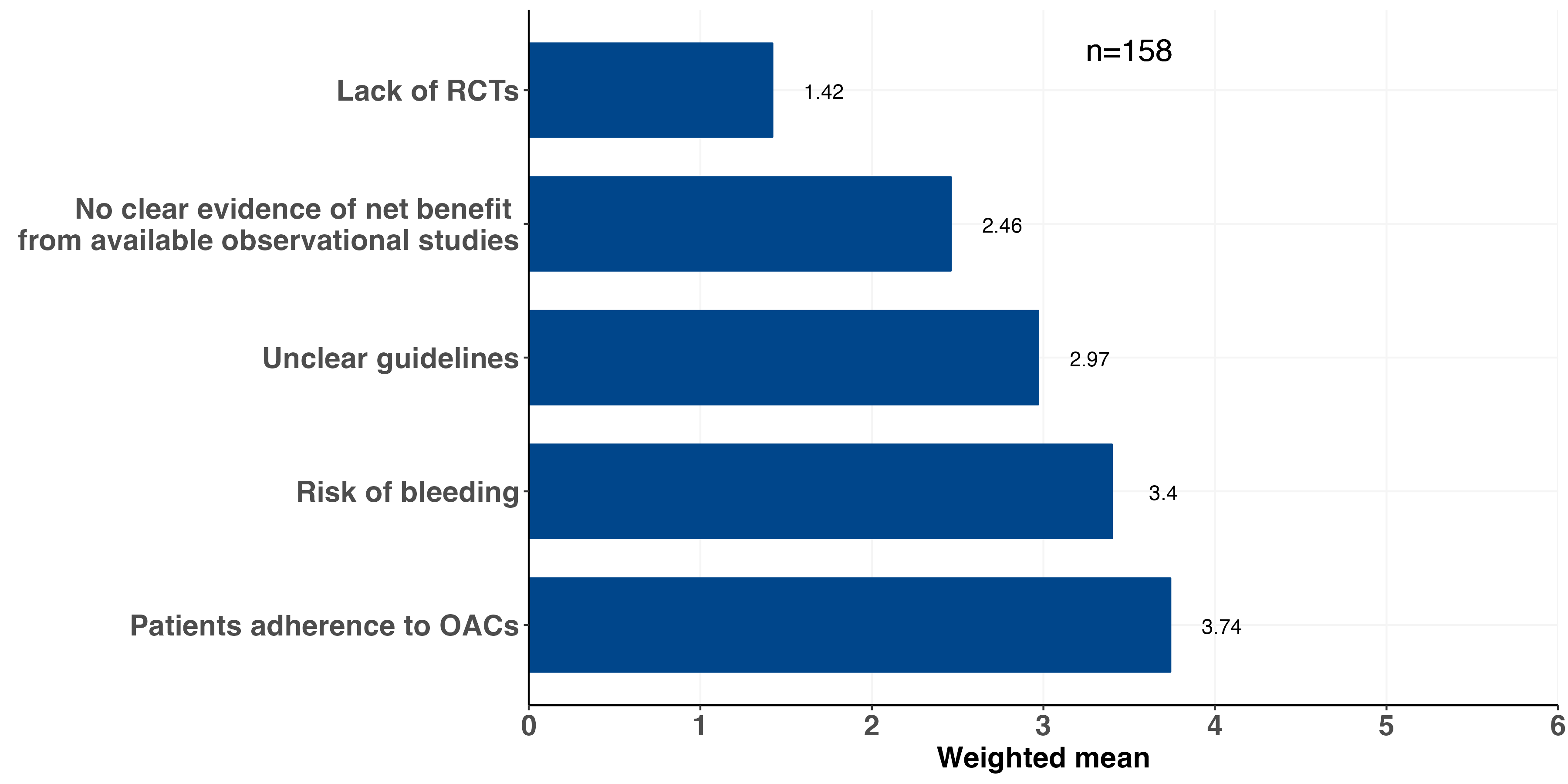


**Supplementary Figure 3.** Involvement of cardiologists in decision making for oral anticoagulants prescription and discharge/follow-up plan in the setting of postoperative atrial fibrillation occurring after coronary artery bypass grafting.

**
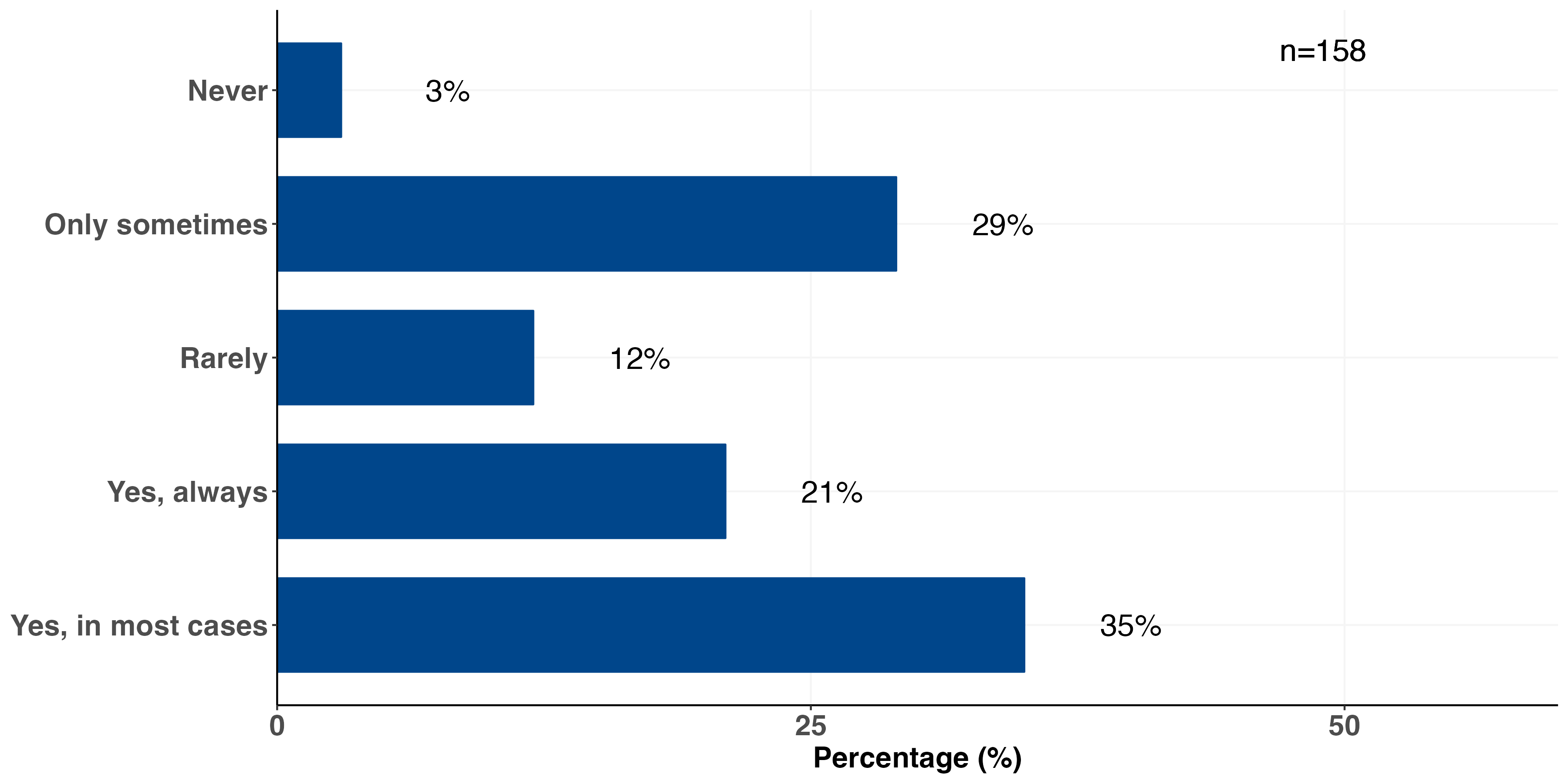
**

**Supplementary Figure 4.** Minimum duration of postoperative atrial fibrillation (POAF) episode required to consider oral anticoagulants prescription, in the setting of POAF after non-cardiac surgery.

H, hours; min, minutes; POAF, postoperative atrial fibrillation; s, seconds.

**
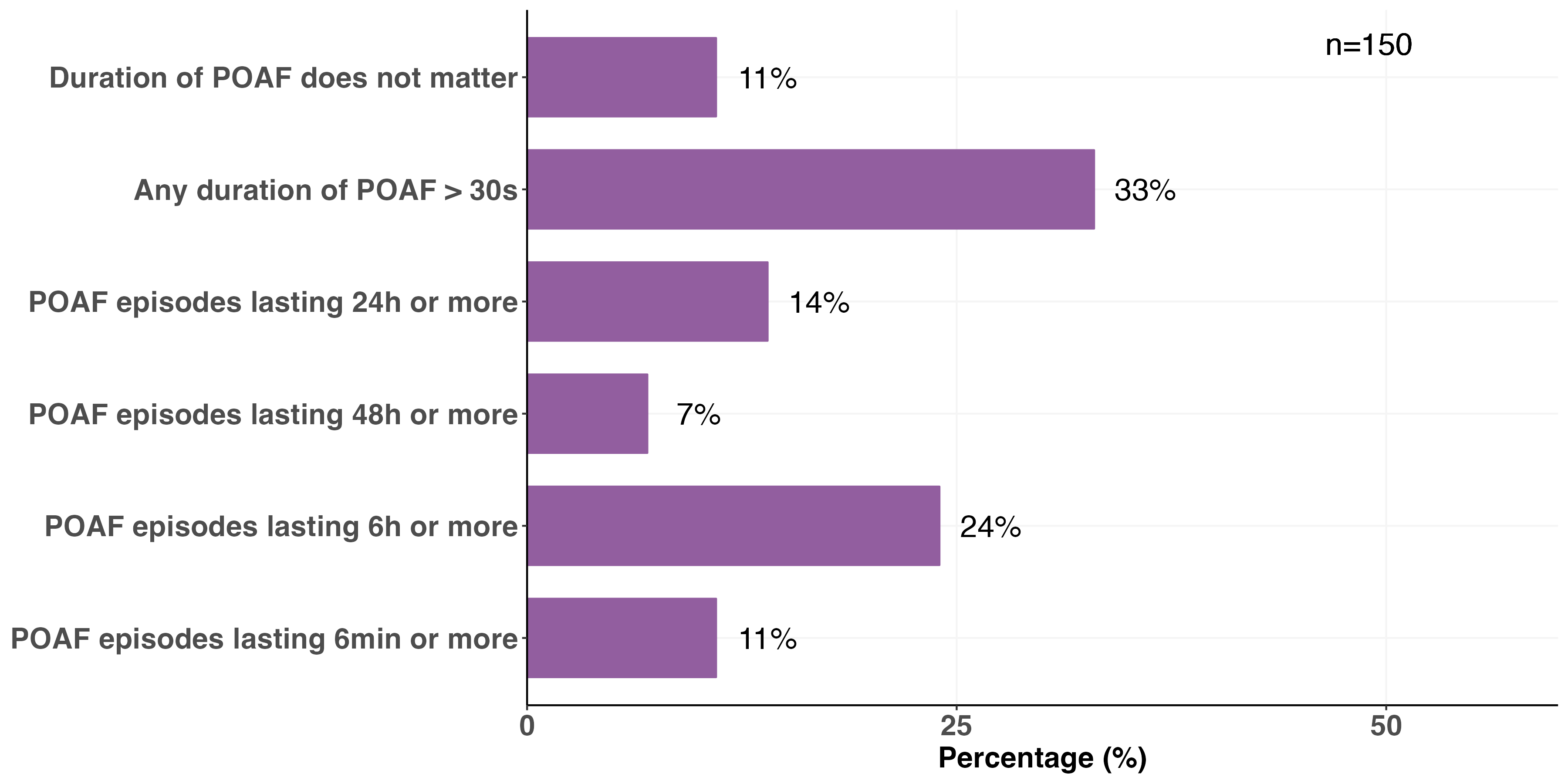
**

**Supplementary Figure 5.** Main issues concerning decisions about long term anticoagulation in patients with postoperative atrial fibrillation in the setting of non-cardiac surgery in patients at increased stroke risk according to CHA_2_DS_2_-VASc/CHA_2_DS_2_-VA.

OACs, oral anticoagulants; RCTs, randomized controlled trials.


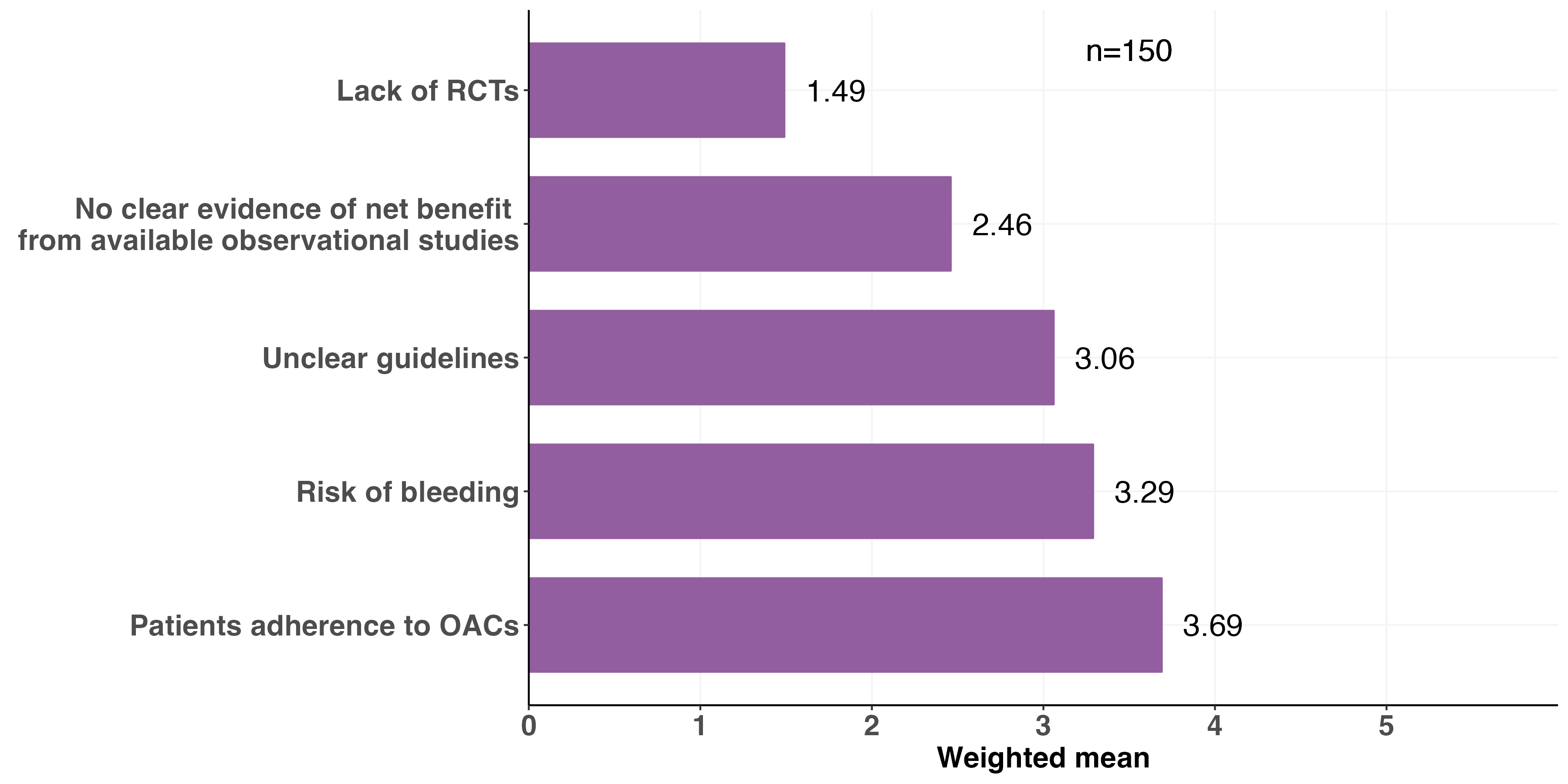


**Supplementary Figure 6.** Involvement of cardiologists in decision making for oral anticoagulants prescription and discharge/follow-up plan in the setting of postoperative atrial fibrillation occurring after non-cardiac surgery.

**
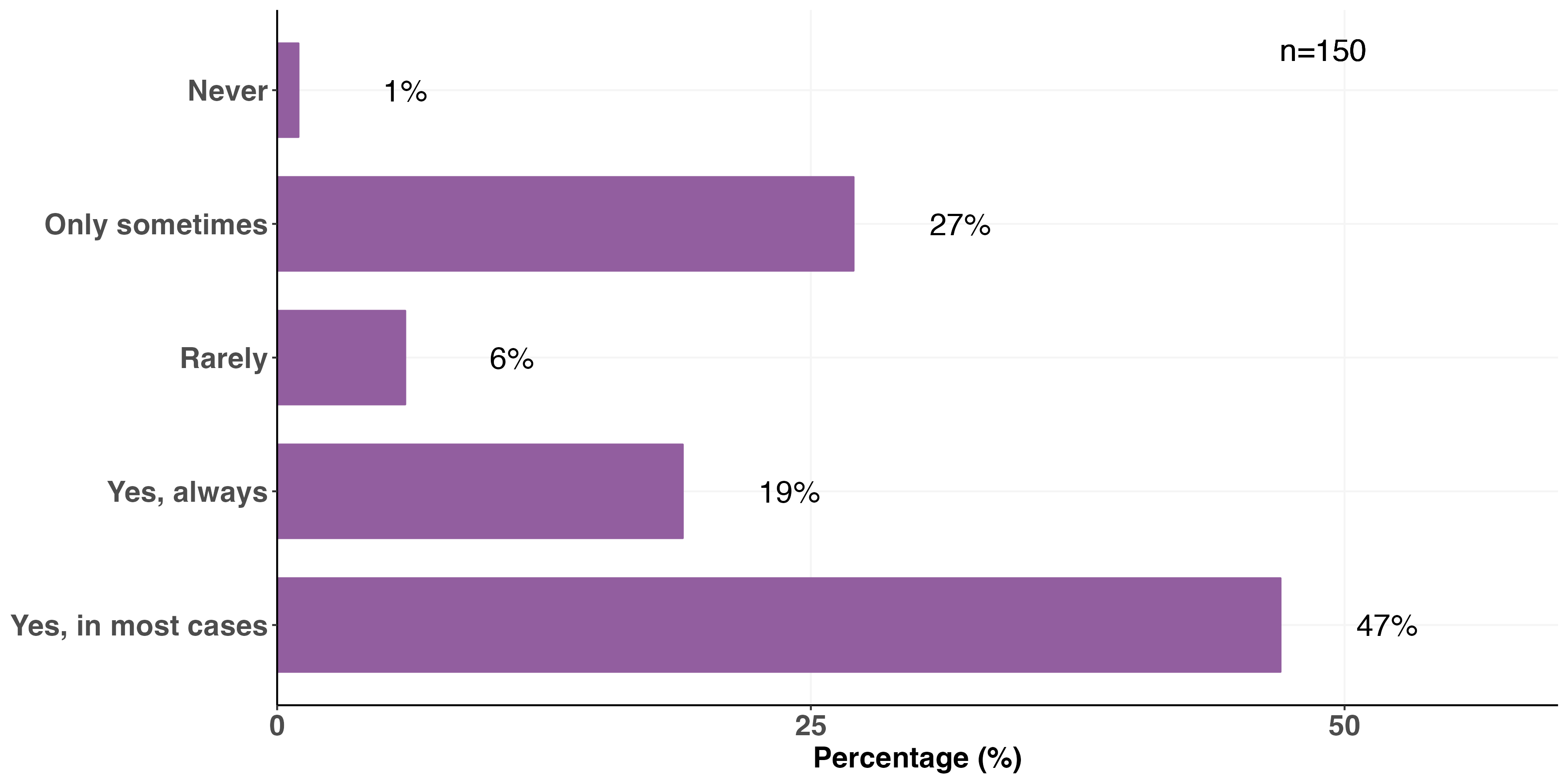
**
